# Surgical outcomes of spinal cavernous malformations: A retrospective study of 98 patients

**DOI:** 10.3389/fsurg.2022.1075276

**Published:** 2023-01-12

**Authors:** Dengyong Liao, Ruoran Wang, Baoyin Shan, Haifeng Chen

**Affiliations:** Department of Neurosurgery, West China Hospital, Sichuan University, Chengdu, China

**Keywords:** cavernous malformation, spinal, surgery, during of symptoms, outcome, hemilaminectomy

## Abstract

**Objective:**

Spinal cord cavernous malformation (SCCM) is a rare vascular lesion, and the treatment strategy remains controversial at present. The goal of this retrospective study was to analyze the surgical outcomes of the SCCM and to find more appropriate treatment strategies for a better prognosis.

**Method:**

A retrospective review of 98 patients with SCCM from 2009 to 2018 was conducted at the neurosurgical center of our hospital. Neurological function was assessed using the American Spinal Injury Association (ASIA) impairment scale. Clinical features were analyzed using the multivariable logistic regression.

**Results:**

Ninety-eight patients with SCCM were included, of whom 36% were female and 64% male. The mean age was 41.6 years; and family history was reported in 8% of patients. Definite hemorrhage was found in 6%. Before surgery, the neurological status was Grade A in 2%, Grade B in 2%, Grade C in 12%, Grade D in 54%, and Grade E in 30% of the patients. 83% (81/98) patients had long-term follow-up, of whom, 42% had improved, 51% were stable and 7% had deteriorated. Patients with dorsal or superficial lesions showed better improvement than those with ventral or lateral deep lesions. Those with symptoms lasting less than three months had higher rates of improvement than those with symptoms lasting more than three months. However, there was no significant difference in prognosis between hemilaminectomy and laminectomy.

**Conclusion:**

These results suggest that surgical strategies should be preferred for severe symptomatic SCCMs if total resection can be achieved, thereby avoiding the risk of severe complications with subsequent lesion hemorrhage. Earlier (usually within 3 months of symptom duration) surgical resection generally may lead to a better prognosis. For ventral or lateral deep SCCMs, the surgical strategy should be considered more carefully.

## Introduction

Cavernous malformations (cms) of central nervous system are rare vascular abnormalities with an incidence of 0.5% in the general population (1, 2) and the genetic basis of cms has been well established (3). Familial cm typically presents with multifocal cms and/or a family history, which is caused by a loss of function mutation in one of three genes, including ccm1 (krit1), ccm2 (mgc4607), and ccm3 (pdcd10). The functions of these genes are all involved in signaling networks that maintain the integrity of the connections between neighboring vascular endothelial cells. Identification of at-risk individuals with cm through pedigree and additional genetic testing can provide counseling for individuals and family members (3).

Most cms occur in the intracranial structures, and very few in the spinal cord. Cms of the spinal cord (sccm) are rarer, accounting for approximately 5%–12% of all spinal cord vascular anomalies (4–6). Compared to intracranial cms, sccms are more aggressive because the spinal canal is less tolerant to space-occupying lesions. The onset time of sccms occurs primarily in the third and fourth decades of life, with a slightly more prominent incidence in women, and the hemorrhage rates are approximately 1.4%–6.8% per lesion and per year (7–9). The preoperative diagnosis of sscm is easier to make due to its typical appearance on magnetic resonance imaging (mri) (10).

Current therapeutic strategies favor the early removal of symptomatic sscm under the microscope once a diagnosis is established. In terms of surgical outcomes, reports indicated that 66% of patients experienced improvement, 28% showed stabilization, and only 50% achieved long-term control or improvement in pain (11). However, for asymptomatic cases diagnosed by chance, conservative strategies or surgical treatment remain controversial, often depending on the preference of the neurosurgeons for this option. At present, several issues are still under debate, such as the choice of conservative treatment and surgical treatment, timing of surgery, surgical approach, and outcomes of surgical resection of sccms.

In this retrospective study, we have detailed the epidemiology, clinical characteristics, surgical modalities and long-term outcomes of 98 patients with sccm treated at our neurosurgical center. We have also analyzed possible predictors associated with better outcomes, with the aim of providing neurosurgeons with additional information when choosing an optimal treatment strategy.

## Materials and methods

### Patient characteristics

The local ethics committee of our institution approved this study and patient consent was obtained from all patients enrolled in this study. We retrospectively analyzed the data of all sccm patients treated at our center from 2009 to 2018 through a prospective maintained database. Clinical characteristics, family history, mri results, and follow-up outcomes were included. Duration of symptoms was defined as the time from onset of symptoms to admission to our hospital. A hemorrhage event was strictly defined as a hematoma found during surgery. The american spinal injury association (asia) impairment scale was used to evaluate the preoperative and postoperative neurological status (12). The asia scale was classified into grades a to e, while grades a to c were defined as severe neurological and disability status, and grades d or e were defined as mild neurological and disability status. All patients underwent preoperative, postoperative, and follow-up spinal mri. The size of the cm was calculated using the maximum diameter measured by the preoperative mri. They were divided into cervical, thoracic or lumbar segment based on the location of the lesion. Lesions in the spinal cord site were classified as ventral or lateral deep and dorsal or superficial. Postoperative mri was used to assess total resection, rehemorrhage and recurrence. 98 surgical patients, of whom 35 women and 63 men, with mean age of 41.6 years (15–80 years), were included in our cohort.

### Surgical information

All patients with sccm underwent surgery by either laminectomy or hemilaminectomy. During surgery, a dorsal midline myelotomy or dorsolateral myelotomy was performed primarily on the discolored surface of the spinal cord, and the lesion was removed microscopically. Somatosensory evoked potentials (ssep) and motor evoked potentials (mep) were monitored during the operation.

### Follow-up outcomes

Postoperative follow-up was classified into short-term (about 3 weeks, before discharge) and long-term (6 months to 5 years). Short-term follow-up information on patients was obtained prior to discharge. Long-term follow-up information (more than 6 months) was collected mainly through outpatient review and telephone interview. Neurological status was divided into improved and unimproved, which included stable or worse outcomes over the long-term follow-up period. Patients whose follow-up was lost were excluded.

### Statistical analysis

All statistical analyses were performed using spss software (version 26.0) and graphpad software (version 8.3.3). *T*-test, pearson's *χ*^2^ test and multivariable logistic regression analysis were used to analyze the factors associated between the improved and unimproved groups (including stable and worsened). In the multivariable logistic regression analysis, age, sex, family history, single or multiple lesions, size, location, site in the spinal cord, involved segment, asia score, hemorrhage, total resection, surgical approach and duration of symptoms were included in the regression model. The statistical significance was *P* < 0.05.

## Results

### Patient characteristics

A total of 98 patients with sccm were admitted to our center between 2009 and 2018. Of whom 36% were female and 64% were male. The mean age was 41.6 years (15–80 years), 4% of the patients had more than one independent lesion occurring in the intracranial or other spinal segments, and 8% had a family history. Of those who present with symptoms, 47% patients (*n* = 46) complained of pain, 54% patients (*n* = 53) of paresthesia, 36% (*n* = 35) of decreased muscle strength and 16% (*n* = 16) of combined bladder dysfunction. The duration of symptoms was 10.0 ± 13.3 months (from 1 day to 5 years). Preoperative hemorrhage was confirmed in 6 cases, and according to mri, 50 lesions were located in the cervical segments, 45 were located in the thoracic segments, and only 3 were located in the lumbar segments. Total resection was performed in 94 patients and subtotal resection was performed in only 4 patients because these 4 lesions were mainly located in ventral of the spinal cord and lacked a clear interface between the lesion and the medulla. The four lesions remained stable and did not hemorrhage during subsequent rigorous follow-up. Of the lesion's site, 66 were located in the dorsal or superficial spinal cord, and 32 were located in the ventral or deep spinal cord. 59 sccms of the patients were resected by hemilaminectomy, while 39 by laminectomy, and the hemilaminectomy-to-laminectomy ratio was 1.5:1. Improvement in patient symptoms during follow-up was seen in Table 1 and the characteristics of the 98 patients are described in Supplementary Table S1.

**Table 1:** Characteristics of the improved and unimproved groups.

Characteristics	Improved (*n* = 34)	Unimproved (*n* = 47)
**Sex**		
Male	19 (56%)	34 (72%)
Female	15 (44%)	13 (28%)
Age (years, mean ± SD)	35.7 ± 14.3	41.5 ± 15.8
Multiple lesions	2 (6%)	2 (4%)
Family history	1 (3%)	4 (9%)
**Clinical signs**		
Pain	16 (44%)	20 (56%)
Sensory deficits	13 (28%)	33 (72%)
Motor deficits	15 (54%)	13 (46%)
Bowel/ urinary defect	8 (62%)	5 (38%)
Duration of symptoms (months)	4.6 ± 7.5	13.3 ± 14.3
**Location**		
Cervical	19 (56%)	22 (47%)
Thoracic	13 (38%)	24 (51%)
Lumbar	2 (6%)	1 (2%)
Involved segments	1.7 ± 0.6	1.6 ± 0.5
Mean size of CMs (cm)	1.2 ± 0.6	1.1 ± 0.4
hemorrhage rate	2 (6%)	3 (6%)
**ASIA scale**		
ASIA A-C (severe)	12 (86%)	2 (14%)
ASIA D-E (mild)	22 (33%)	45 (67%)
**Surgical resection**		
Totally removed	33 (43%)	44 (57%)
Sub totally removed	1 (25%)	3 (75%)
**Lesions in the spinal cord**		
Dorsal or superficial	29 (54%)	25 (46%)
Ventral or lateral deep	5 (19%)	22 (81%)
**Surgical approach**		
Laminotomy	14 (42%)	19 (58%)
Hemilaminectomy	20 (42%)	28 (58%)
Follow-up (months)	33.6 ± 13.2	33.8 ± 11.5

### Neurological function preoperatively and postoperatively

The neurological status of all patients was assessed with the ASIA scale before surgery (Table 2). Both short-term (1–3 weeks) and long-term (over 6 months) postoperative outcomes were assessed according to the same scale. An increase in grade from A to E was defined as an improvement, and remaining the same or a decrease in grade was defined as a stabilization or deterioration, respectively. Preoperatively, the neurological status was Grade A in 2%, Grade B in 2%, Grade C in 12%, Grade D in 54%, and Grade E in 30% of the patients.. In the short-term follow-up (*n* = 98), the proportion of grade A was disappeared, while the proportions of grade B and C increased to 4% and 28%, respectively, and the proportions of grade D and E decreased to 52% and 16%, respectively (Table 3). During short-term follow-up, the patients of grade A and B improved or stabilized postoperatively; one patient with grade C worsened and 11 improved or stabilized; 11 and 13 patients of grade D and E deteriorated, while 35 and 16 patients preserved stable or improved, respectively (Table 4). During long-term follow-up, a total of 81 patients were assessed with the ASIA scale, and 17 were lost to follow-up. The mean duration of follow-up was 34 months (6–60 months). As shown in Tables 3, 4, better surgical outcomes could be seen compared to preoperative and short-term follow-up. Patients with grades A, B, C and D showed significant decreases, while those with grades E showed increases. Specifically, over long-term follow-up, a total of 75 patients improved or remained stable and only 6 patients deteriorated (Table 4).

**Table 2:** American spinal injury association (ASIA) impairment scale.

Grade	Details
A	No sensory and no motor function is preserved in sacral segments S4–S5.
B	Sensory function is preserved but motor function is not preserved below the neurological level including the sacral segments S4–S5.
C	The preservation of motor function is below the neurological level, and more than half of key muscles have the muscle grade less than 3 below the neurological level.
D	The preservation of motor function is below the neurological level, and at least half of the key muscles have a muscle grade of 3 or more below the neurological level.
E	Sensory and motor function are normal.

**Table 3:** Preoperative and postoperative neurologic Status according to ASIA.

Grade	Preoperative (*n* = 98)	Short-Term Outcome, 1–3 Weeks (*n* = 98)	Long-Term Outcome, 6–60 months (*n* = 81)
A	2 (2%)	0	0
B	2 (2%)	4 (4%)	0
C	12 (12%)	27 (28%)	8 (10%)
D	53 (54%)	51 (52%)	40 (49%)
E	29 (30%)	16 (16%)	33 (41%)

**Table 4:** Postoperative neurological improvement of the patients.

Status	ASIA scale					
	Total	A	B	C	D	E
Preoperative	98	2	2	12	53	29
Postoperative short-term outcomes	98					
Worsened	32			1	18	13
Stable or improved	66	2	2	11	35	16
Postoperative long-term outcomes	81					
Worsened	6				5	1
Stable	41			2	31	8 (pain)^a^
Improved	34	2	2	8	9	13 (pain)^a^
Lost to follow-up	17			2	8	7

^a^ Because there is no better condition than grade E by ASIA. We assigned grade E patients into the “stable” (no improvement in pain) or “improved” (pain relief or disappearance) conditions during long-term following up.

### Factors predicting improved and unimproved groups

During the long-term follow-up, patients with the same or decreased ASIA grade were divided into an unimproved group and those with increased ASIA grade into an improved group. Because patients in grade E mainly presented with pain symptoms and there was no better status than grade E according to ASIA scale, patients in grade E with disappearance or marked relief of pain were divided into the improved group, whereas patients with no improvement or worsening of pain were classified into the unimproved group. Therefore, a total of 81 patients were finally included during the long-term follow-up, of whom 34 showed improvement in neurological status or pain symptoms and 47 showed no improvement of these. Motor weakness improved significantly, whereas of the 46 patients with sensory deficits, only 13 improved. 14 patients with severe preoperative neurological dysfunction (ASIA A-C), of whom 12 had significant improvement, and 2 had no improvement. Among 67 patients with mild preoperative neurological impairment (ASIA D-E), only 22 of the patients had improved symptoms, and 45 patients had no improvement. The lesions located in the dorsal or superficial of the spinal cord had better improvement with a percentage of 54% (*n* = 29), while the improvement rate of the ventral or lateral deep lesions was 19% (*n* = 5). There was no significant difference between hemilaminectomy and laminotomy (*P* = 0. 946) (Table 1).

Referring to previous studies on SCCMs (13), we used multivariable logistic regression analysis to find predictive factors that might be associated with better prognosis (Table 5). As shown in Table 5, there were no statistically significant differences between the two groups in terms of age, sex, single or multiple lesions, family history, SCCM size, hemorrhage, preoperative ASIA grade, totally resected, and hemilaminectomy or laminotomy (*P* > 0.05). However, there were statistically significant differences (*P *< 0.05) between the improved and unimproved groups in terms of sensory disturbance, duration of symptoms, and lesions located dorsal or superficial and ventral or lateral deep of the spinal cord.

**Table 5:** Multiple logistic regression analysis between improved and unimproved group.

Status	*P* value	OR	95% confidence interval
Age	0.132	1.043	0.987–1.101
Sex	0.766	1.295	0.236–7.101
Multiple lesions	0.336	0.058	0.000–19.108
Family history	0.156	10.770	0.403–287.806
Sensory deficits	0.006	10.860	1.965–60.008
Duration of symptoms (months)	0.007	1.154	1.040–1.280
Mean size of CMs	0.426	0.531	0.112–2.523
Hemorrhage	0.998	1.385E + 10	–
ASIA scale	0.998	1.559E + 11	–
Surgical resection	0.863	1.568	0.022–110.217
Lesions in the spinal cord	0.011	0.067	0.008–0.540
Surgical approach	0.105	0.271	0.056–1.316

## Discussion

To date, the available data for patients with SCCM are still insufficient due to the low incidence of SCCM (4, 14, 15). The current debate focuses on two points: the natural history of SCCM and conservative vs. surgical management (4, 16). In previous studies, numerous authors have described a slight predominance of females in the SCCM, at a ratio of 1:1.1 (17, 18), with the highest incidence commonly occurring in the third and fourth decades of life (19). The mean age of the SCCMs in our series is 41.6 years, which is close to the results of previous studies (14, 20). However, the rate for men and women was 1:0.56, which was different from previous studies. The reason might be that the samples in our study were all surgical patients, selection bias was a contributing factor for this difference. Early SCCMs are usually asymptomatic or only transiently symptomatic with minor numbness or pain, and are often easily overlooked by patients due to memory bias, whereas some patients only have incidental detection of SCCMs on a systemic examination, resulting in a bias in the duration of symptoms. Thus, it is difficult to investigate the natural history of the SCCM. SCCM haemorrhage is defined as a hematoma found during surgery, including cases that were not found haemorrhage on preoperative MRI. It is well recognized that the common clinical feature of intramedullary CM is slowly progressive neurological deterioration, which appears to be associated with several minor hemorrhages (21). Symptoms caused by a small amount of bleeding may be extremely mild and the patient does not feel a noticeable change in symptoms. Moreover, the haemorrhage rate is mainly based on surgical cases, ignoring bleeding from CMs in conservatively treated patients. As a result, the annual haemorrhage rate of SCCMs may be significantly higher than actually reported. A series of previous studies reported annual hemorrhage rates of 1.6%–4.5% (20, 22, 23), and our study had a bleeding rate of 6%, slightly higher than reported. Badhiwala et al. reported that approximately 61% of patients had motor impairment, 58% had sensory impairment, 34% had pain, and 24% had bladder and/or bowel impairment (14). Of our 98 cases, 36% of patients experienced motor dysfunction, 54% experienced sensory impairment, 47% experienced pain, and bladder and/or bowel disorders accounted for approximately 16%. The reason for the discrepancy between these results and the previous literature may still be related to the finite sample size. The same is true for family history and multiple lesions. In our series, eight patients (8%) had a family history and four patients (4%) had multiple lesions, while Badhiwala's meta-analysis showed that 12% had a family history (14), and 17 cases (34%) were combined with intracranial CMs as reported by Mitha et al. (24). One possible reason is that not all patients and family members are willing to undergo a full MRI examination.

For asymptomatic or mild symptomatic SCCMs, however, which are particularly located deep in the ventral or lateral portions of the spinal cord, surgery can result in serious complications such as paralysis, and the choice of conservative treatment or surgical treatment is still another controversial. Kharkar et al. (9) reported on 14 patients with symptomatic SCCMs managed conservatively, and 71% (*n* = 10) were clinically stable at a mean follow-up of 80 months. Although these findings establish the benefits of conservative treatment, the number of cases is too small and additional samples are needed to further investigate the benefits and risks of conservative treatment. Numerous conservatively treated patients have asymptomatic or mild symptoms, and such patients tend to be inactive during clinical follow-up because their symptoms have little impact on daily life, resulting in a substantial lack of follow-up data. In addition, some patients frequently actively seek surgical treatment once SCCM is diagnosed, and neurosurgeons have a preference for surgical treatment. It has been reported that the majority (90%) of patients with SCCMs underwent surgical resection, while only 10% received conservative treatment (14). For these reasons, the number of conservatively treated patients is limited and the corresponding studies are few. As a result, the lack of research on the effects of conservative treatment may lead to a bias against the benefits of surgical treatment.

For most patients with symptomatic SCCM, surgical removal remains an effective treatment to eliminate the associated lifetime risk of hemorrhage. Badhiwala et al. showed that symptoms improved in 51% of patients, remained the same in 38% and worsened in 11% during long-term follow-up (14). In our study, 83% patients (81/98) underwent long-term follow-up, which was within the acceptable range for data analysis. Of these, 42% showed improvement in symptoms, 51% remained stable and 7% deteriorated. This result compares unfavourably with previous data, but we found that in our study there was a higher proportion of patients with only pain and sensory disturbance, both of which were the most difficult to improve with surgery. The literature reports improvement rates of about 50% for postoperative pain and only 7% for sensory disturbances (25, 26). Bian et al. proposed that motor abnormalities had a more favorable clinical outcome than sensory symptoms (20). Park et al. showed that after complete resection, sensory deficits generally persisted during long-term follow-up (26). Our study also found that although there was no significant correlation between overall symptoms and prognosis in the multivariable regression analysis, there was a significant difference between the improved group and the unimproved group of sensory impairment (*P* < 0.05), indicating that sensory function was more difficult to recover than motor function. The literature suggests that the dorsal column is more susceptible to dorsal or dorsolateral myelotomy than the ventral or lateral motor fibers, which contributes to poor improvement in sensory deficits (26). Moreover, macrophages, which are abundant in hemosiderin in the post-surgery lesion, may additionally damage the sensory tract, causing the patient to suffer from paresthesia (26). Interestingly, in univariate analysis, we found that patients with severe symptoms (ASIA A-C) had a significantly better symptom improvement rate than patients with mild symptoms (ASIA D-E) (*P* < 0.001). Among the 14 patients with severe neurological dysfunction at follow-up, 12 patients (86%) showed better functional status, and two patients (14%) showed no improvement. The reason for this result may be related to the shorter duration of symptoms in patients with severe neurological disorders. We also found that patients with mild symptoms (Asia D-E) had a higher rate of symptom deterioration in the short postoperative period, but most of these patients recovered during long-term follow-up, only 6 patients had worsened, mainly manifested as numbness or increased pain without severe functional disability such as paralysis (Table 4). These data suggest that timely surgical resection could effectively improve the neurological status of patients with severe preoperative conditions. This is also consistent with the literature reports (25). These conclusions have important implications for treatment decisions. If the patient has only minor pain or sensory deficits that do not affect their quality of life, conservative treatment may be reasonable, as surgical outcomes for sensory deficits are unsatisfactory. However, if the sensory deficit is severe, aggressive surgical treatment should be recommended.

Based on long-term follow-up data, we analyzed the characteristics of the improved and unimproved groups. The data showed that the overall improvement rate was about 42% (34/81), which was close to the results of Badhiwala's systematic review (14). Reports suggest that the duration of symptoms is an important predictor of outcome. Cantore et al. qualitatively observed that patients with a longer clinical history had a worse prognosis (27). In recent years, a larger retrospective analysis has shown that patients with symptoms that last less than three months have higher rates of improvement than those with symptoms that last longer (28). A similar conclusion was found in our study, where the duration of symptoms was 4.6 ± 7.5 months of the improved group compared with 13.3 ± 14.3 months of the unimproved group (*P* < 0.0018), with a statistically significant difference. Based on these data, we do recommend that severe symptomatic SCCM should be treated surgically as early as possible, preferably within 3 months, which may lead to better clinical outcomes. Several studies have found that the severity of preoperative neurological injury is associated with poor outcomes (8). In contrast, patient age, sex, family history, multiple lesions, involved segments, and hemorrhage were not associated with prognosis (28).

We also found that the improvement rate of ventral or lateral deep SCCMs was lower than that of dorsal or superficial SCCMs, and the difference between the two groups was statistically significant (*P* = 0.011). The reason may be that ventral or lateral deep SCCMs are more difficult to remove, resulting in greater intra-operative damages to the spinal cord. Therefore, some authors have suggested that in the absence of symptoms or mild symptoms, close follow-up rather than surgical resection may be recommended for ventral or lateral deep lesions (18, 20) However, rigorous follow-up is necessary when choosing conservative treatments. They have also suggested considering a switch to surgical treatment when the MRI shows an increase in lesion size, especially if the surface of the spinal cord is raised in the posterior position (18, 20).

In recent years, with the development of microsurgical techniques and various positioning devices, some authors have become more and more in favor of resection of intramedullary lesions by hemilaminectomy (20, 29–33). We also analyzed the data of laminectomy and hemilaminectomy, and the rate of hemilaminectomy was 59% (48/81). There was no significant difference in symptom improvement between these two surgical procedures. This finding may be attributed to smaller iatrogenic spinal cord injury, milder postoperative reactions, and better spinal stability associated with minimally invasive microsurgery (30, 34–36).

The main limitation of this retrospective study is the inherent selection bias associated with the single-centre retrospective nature of the data and the relatively small sample size. This problem stems from the extreme scarcity of SCCM, limiting the ability to conduct prospective studies and/or randomized trials. Therefore, there is an inherent selection bias in this study that should be carefully considered when interpreting the results. In addition, all the patients have undergone surgery, however no data were available for conservatively treated patients, so future studies are needed to develop new therapeutic strategies to administer asymptomatic or mild symptomatic SCCMs.

## Conclusion

For SCCM with severe symptoms, our results suggest that surgical therapy should be considered as the first choice. Early microsurgical resection might improve the prognosis of patients with neurological deficits. Since postoperative improvements in only pain and/or sensory deficits tend to be less satisfactory, conservative treatment also seems to be an potential option for these patients. Especially for CM located in the ventral or deep lateral spinal cord, surgical treatment should be more carefully considered. Hemilaminectomy and laminectomy are not prominent prognostic factors in our study. Therefore, minimally invasive hemilaminectomy may be preferred for this procedure in some situation.

## Data Availability

The raw data supporting the conclusions of this article will be made available by the authors, without undue reservation.
