# Supplementary material for: Surgical outcomes of spinal cavernous malformations: A retrospective study of 98 patients
Source: Front Surg. 2023 Jan 12;9:1075276. doi: 10.3389/fsurg.2022.1075276 (PMC9877401; doi:10.3389/fsurg.2022.1075276)
Supplement: Supplementary file 1 [file Table1.docx]

**Supplemental** **Table 1: General Characteristics of patients**

| **Characteristics** | **Patients (n=98)** |
| --- | --- |
| Sex |  |
| Female (n, %) | 35 (36%) |
| Male (n, %) | 63 (64%) |
| Age (years, mean±SD) | 41.6±16.7 |
| Multiple lesions (n, %) | 4 (4%) |
| Family history (n, %) | 8 (8%) |
| Clinical signs |  |
| Pain (n, %) | 46 (47%) |
| Sensory deficits (n, %) | 53 (54%) |
| Motor deficits (n, %) | 35 (36%) |
| Bowel/ urinary dysfunction (n, %) | 16 (16%) |
| Duration of symptoms (months) | 10.0±13.3 |
| Location (n, %) |  |
| Cervical | 50 (51%) |
| Thoracic | 45 (46%) |
| Lumbar | 3 (3%) |
| Involved segments (mean±SD) | 1.7±0.5 |
| Mean size of CMs (cm) | 1.2±0.5 |
| hemorrhage rate (n, %) | 6 (6%) |
| Surgery information |  |
| Totally removed | 94 (96%) |
| Sub totally removed | 4 (4%) |
| Dorsal or superficial lesions | 66 (67%) |
| Ventral or lateral deep lesions | 32 (33%) |
| Laminotomy | 39 (40%) |
| hemilaminectomy | 59 (60%) |
| Follow-up (months) | 34 (6-60) |
